# Managing the consultation with patients with medically unexplained symptoms: a grounded theory study of supervisors and registrars in general practice

**DOI:** 10.1186/s12875-014-0192-7

**Published:** 2014-12-05

**Authors:** Louise Stone

**Affiliations:** Centre for Values, Ethics and the Law in Medicine, University of Sydney, Level 1 Medical Foundation Building, 92-94 Parramatta Road, Camperdown, NSW 2041 Australia; Academic Unit of General Practice, Australian National University Medical School, College of Medicine, Biology and Environment, Australian National University, Canberra ACT, 0200 Australia

**Keywords:** General practice, Therapeutic relationship, Mental health, Somatoform disorders, Medical education, Consultation dynamics

## Abstract

**Background:**

Patients with medically unexplained symptoms (MUS) commonly present in general practice. They often experience significant disability and have difficulty accessing appropriate care. Many feel frustrated and helpless. Doctors also describe feeling frustrated and helpless when managing these patients. These shared negative feelings can have a detrimental effect on the therapeutic relationship and on clinical outcomes. The aim of this study was to explore how novice and experienced GPs manage patients with MUS and how these skills are taught and learned in GP training.

**Methods:**

A constructivist grounded theory study with 24 general practice registrars and supervisors in GP training practices across Australia.

**Results:**

Registrars lacked a framework for managing patients with MUS. Some described negative feelings towards patients that were uncomfortable and confronting. Registrars also were uncertain about their clinical role: where their professional responsibilities began and ended. Supervisors utilised a range of strategies to address the practical, interpersonal and therapeutic challenges associated with the care of these patients.

**Conclusions:**

Negative feelings and a lack of diagnostic language and frameworks may prevent registrars from managing these patients effectively. Some of these negative feelings, such as frustration, shame and helplessness, are shared between doctors and patients. Registrars need assistance to identify and manage these difficult feelings so that consultations are more effective. The care of these patients also raises issues of professional identity, roles and responsibilities. Supervisors can assist their registrars by proactively sharing models of the consultation, strategies for managing their own feelings and frustrations, and ways of understanding and managing the therapeutic relationship in this difficult area of practice.

## Background

Patients with medically unexplained symptoms commonly present in primary care [1,2]. Many of these patients have significant functional impairment [3,4] with similar disability to patients with depression or anxiety [5–7]. They are often “frequent attenders”: [8,9] contributing significantly to a GP’s workload over a prolonged period of time [10].

These patients often experience stigma and shame [11]. Without a diagnosis, they lack a coherent narrative to make sense of their symptoms [12]. Many have also experienced early childhood trauma and have interpersonal difficulties which make it challenging to develop and maintain trusting therapeutic relationships [13].

Consultations around medically unexplained symptoms are often unsatisfying and difficult for doctors and patients [14–19]. When patients experience distressing symptoms, and doctors cannot provide a diagnosis that validates their suffering, there is the potential for significant conflict in the consultation. The result is often “a duet of escalating antagonism” [20] a consultation which various writers have described as a law court, a medieval siege or a tug of war [11,21]. While effective consultations involve co-constructed meaning, with patients drawing on illness schema and doctors drawing on disease schema [22], in these consultations, meaning is contested or confused [23]. It is therefore understandable that doctors can find these patients frustrating and difficult to help [24–27]. The use of terms such as “difficult” [27] “hateful” [28] or “heartsink” [29] reflects the way negative emotions can be triggered in the doctor.

Most doctors recognize the importance of the therapeutic relationship, and feel responsible for it even when it is difficult [30]. However, they fluctuate in their willingness and capacity to engage with patients’ emotional cues [31]. Doctors often feel overwhelmed by the task of caring for patients with medically unexplained symptoms [15,26,32,33], and lack confidence in their ability to meet patients’ needs [34–36]. Some distance themselves from their patients as a way of managing their own difficult feelings [37,38]. Negative emotions, such as frustration or a sense of helplessness, can also be shared between doctor and patient. Kirmayer calls these “looping effects”: vicious cycles where the emotion of one person can trigger heightened feelings in the other and escalate difficult emotions in both [39].

There are also issues around diagnostic classification. Although many patients with medically unexplained symptoms share common features, shaping these symptom clusters into a disorder category has been difficult [40,41]. There are currently a number of ways of conceptualising their symptomatic distress [42]. Despite the efforts of an international working group in the preparation of DSM 5, there is still significant disagreement about the best way to classify these symptoms into common disorders [43,44]. The various psychiatric disorders that have been proposed or utililsed have limitations [45]. Some are thought to be over-inclusive, others too restrictive [46] and most are limited by the stigma attached to the diagnostic terms [47]. It is therefore not surprising that patients with medically unexplained symptoms often present a confusing picture for the general practitioner.

Despite these difficulties, several studies have suggested that GP training can be effective in improving outcomes for patients with medically unexplained symptoms [48–52] although some studies suggest this effect is small [53], and not sustained [54]. There is some evidence for the improvement of patient outcomes with other interventions involving GP training, particularly involving a technique known as reattribution [53,55–59] but a recent narrative review suggests existing models of training may be too simplistic to meet the needs of these complex patients [60].

The aims of this study included exploring the reasoning processes utilised by novice and experienced GPs when assessing and managing patients who present with medically unexplained symptoms. The researchers also aimed to understand how these skills are taught and learned in the context of the GP training practice environment and the professional relationship between supervisors and registrars. Grounded theory methodology was used to try to conceptualise the process of the consultation as it is understood by novice and experienced GPs.

## Methods

### Context

General practice training in Australia occurs in one of 17 regional training providers, under the supervision of a Director of Training. Registrars commence GP training following at least one postgraduate year of generalist hospital training. Training consists of placement in a series of general practices with GP supervisors, who provide in-practice teaching and clinical supervision and external educational activities and peer learning provided by the medical educators of the regional training provider.

### Theoretical perspectives

This study is grounded in the symbolic interactionism tradition with its fundamental assumption that reality and the self are known through interaction and expressed through communication and language [61,62]. This perspective is important in this study on several levels. The GP consultation, particularly when it focusses on psychosocial concerns, relies on the dynamic interchange between doctor and patient which leads to interpretation of experience and the construction of meaning. These meanings are changeable: they differ between the two people in the encounter and over time. GPs rely on continuity of care to help them refine their understanding: re-checking and reshaping their ideas, explanations and models as their understanding of the patient’s experience deepens. The process of using interviews for this study drew on this common cultural experience, the construction of meaning between two people using language and conversation.

The supervisor-registrar learning environment also centres on conversation. Although some consultations are observed, most learning occurs when the learner reflects on a consultation, re-enacts or describes the difficulties or challenges with the supervisor, and creates new meanings and interpretations over time. This study utilised interviews, relying again on the conversation between interviewer and interviewee to construct an understanding of clinical processes.

### Study design

The study utilizes Charmaz’s constructivist grounded theory methodology [62] using semi-structured interviews as a research method. Data were collected and analysed iteratively, and during the course of the study models of the consultation emerged and were tested and refined. Interviews were conducted face to face or by telephone and were 45 to 60 minute in duration. All interviews were undertaken by the same interviewer and were transcribed verbatim. Participants and their patients were anonymised and pseudonyms are used throughout the paper.

Interviews began with the participant describing a case of a patient with medically unexplained symptoms. The interviewer then utilised the case to explore the clinical reasoning process, the emotional issues raised and the consultation structure utilised. Registrars were asked to describe how they sought help with the case, and the ways in which they extended their knowledge and skills. Supervisors were asked how they would assist a registrar managing a similar case, and the sorts of difficulties they would expect registrars to experience. All participants at the close of the interview were asked how their thinking had changed through the course of the interview.

### Sampling

Registrars were recruited through convenience sampling using flyers at training workshops. Supervisors were invited to participate, each chosen purposively to challenge and refine emerging theoretical concepts. Sampling was continued until no further analytic concepts emerged from the data.

Supervisors, as a cohort, were known to the researcher due to her extensive experience in medical education in Australia. Supervisors were approached individually, on the basis of their known expertise. Only one supervisor declined to be interviewed, due to overseas travel at the time of the study. Supervisors were chosen as the expert group because of their professional expertise and specific competencies in teaching. These competencies imply that these GPs are both expert and able to articulate their clinical reasoning and clinical processes. The sample was chosen to provide a breadth of perspectives and practices, so that emerging analytic models were likely to be broadly transferable. Sampling was continued until no further analytic concepts emerged from the data.

Eight registrars and sixteen supervisors were interviewed. Characteristics of the sample are detailed in Table 1.

**Table 1:** **Characteristics of the study sample**

**Characteristic**		**Number of participants**
Role	Registrars	8
	Supervisors	16
Sex	Female	11
	Male	13
Age	20-30	4
	30-40	4
	40-50	8
	50-60	6
	60+	2
Context	Urban	12
	Rural	8
	Remote	3
	Aboriginal Medical Service	3
	Correctional facilities	1
Identified interest in mental health	Yes. Sets aside specific consultations for counselling	3
	Yes. Incorporates counselling into their normal consultations	9
	No. Identifies other interests (eg sports medicine, procedural practice)	12

Registrars had 3 to 18 months of GP experience. Supervisors had between 20 and 40 years of general practice experience. All had more than 10 years’ experience as a supervisor of GP registrars.

### Analysis

Data included the interview transcripts, theoretical memos and fieldnotes. Initial transcripts were analysed using line by line coding. As the study progressed, categories emerged from the codes and the categories and their relationship to each other were explored in later interviews. Constant comparative methods were used to compare data within interviews and concepts and processes between interviews. Categories were then explored using analytical memos, which formed the basis for later theory development. Analysis ceased when no further theoretical concepts emerged from the data. Diagrams and mind maps were used to compare the consultation processes described in my data. Over time, descriptions of successful consultations were developed and compared with consultations that became mired in uncertainty and frustration.

### Reflexivity

I am a GP and medical educator with a clinical and teaching interest in mental health and conducted all the interviews. I was not in a direct teaching or supervision relationship with any participants. Some supervisors were known to me, but as they were experienced practitioners, I felt they could be contacted directly and choose not to participate freely.

I was very aware throughout the interviews that my context influenced my decisions and the participants’ perceptions. To quote Charmaz “we are not passive receptacles into which data are poured” [62] p 15. I began the study with a series of what Blumer would call “sensitising concepts”, acquired through years of clinical practice and medical education. I was very aware that there was a common cultural expectation that patients with medically unexplained symptoms were frustrating, demanding and “heartsink”, but that most GPs I worked with educationally felt their care fell firmly within the remit of the GP. I knew that many GPs were deeply committed to biopsychosocial, patient-centred care, but that there were others in the profession who questioned whether a GP needed to take on such broad and ill-defined roles. Our profession has an explicit commitment to continuous, patient-centred, culturally safe care, but it would be naïve to assume all GPs shared these values, demonstrated mastery of the necessary skills, or worked in environments where these values could be readily enacted. Clinical practice has demonstrated to me that many patients experience invalidating, marginalising and stigmatising treatment at the hands of the profession, while others describe supportive and warm therapeutic relationships that are highly valued.

As an educator, I expected registrars to struggle with the complexities of uncertainty. I know that registrars emerge from the tertiary training environment poorly equipped to manage it. I expected in this study for them to continue to search for an elusive diagnosis, that “will reveal itself someday if pummeled by the scientific method” [63] p2398.

My approach to managing these preconceptions was to continue to memo before and after each interview: to capture what I expected to happen, and compare it against the emerging data. I tried throughout to explicitly challenge these preconceptions during the analysis. I have followed grounded theory principles in validating emerging concepts through a process of testing and refining in successive interviews. My approach to theoretical sampling involved choosing participants most likely to challenge my thinking and emerging models.

The most challenging aspect of reflexive practice, however, was not my preconceptions, it was the expectations of the participants. I had not considered the effect of an “expert” researcher asking a GP about their approach to a difficult clinical problem. Every GP has a rich history of being questioned and challenged at the hands of examiners, senior colleagues and clinical supervisors. It was inevitable that the experience of being interviewed would trigger defensive feelings in some participants.

For the registrars, many of these experiences are still raw: I had a number of them share their stories of hospital-based learning, where questions were discouraged, support was elusive and at times they faced painful, challenging experiences alone without an opportunity to debrief. In the interviews, I found I needed to spend a lot of time developing rapport and demonstrating empathy, validating the participant’s experience, before they were prepared to be open and honest about their thinking, and importantly, their feelings. Many did not expect their thinking to be valued, and needed reassurance, both explicit and implicit before they were prepared to be open about their clinical practice. Registrars spoke frankly of their feelings of inadequacy. I was aware of these vulnerabilities and had to be careful to explicitly validate opinions and choices, without forcing the interview down predetermined paths. I checked at the end of each interview what the effect of the process had been, to make sure I had helped them explicate their own thinking, and not imposed my own. I found this question invaluable in helping me reflect on my interviewing technique.

### Ethics

Ethical clearance was provided by the Sydney University Human Research Ethics Committee, (HREC 12269). All participants in the study gave informed consent prior to the interviews.

## Results

The three aims of the study were to explore the reasoning processes utilised by supervisors and registrars with patients with medically unexplained symptoms, to understand the teaching and learning strategies employed by registrars and supervisors in this context, and to model the consultation process. The following section explores each of these aims in detail.

### Exploring the reasoning process

#### Values and validation

The GPs in this study were very aware that their patients were suffering: they discussed how patients felt marginalized and dismissed by the health system. Supervisors expressed a strong ethical commitment to care for the whole patient, even when they found it personally challenging.

*“I think somebody described her as a large demanding blob. She just sits there and is very dependent…And so she was exhausting everybody. Everybody has that same sense of frustration and annoyance with her. And I hate that reaction! I tried so hard not to judge her, and if I walk away, I’d be adding to the people who make her feel that she’s useless, and not worth caring about.”****(Paula –supervisor)***

The GPs in this study felt many patients needed explicit validation, reinforcing that they had a legitimate right to access care. For the registrars, this meant learning to consciously choose to provide supportive treatment in the absence of a diagnosis.

*“The fact is she’d been feeling dismissed by everyone: “The psychologist can’t fix me. The specialists can’t fix me. No one wants me. They all think it’s someone else’s problem.” So I, the first thing I did was I made it my problem. I said, ‘Well, I look after all these things, and so let’s work this out together.’ “****(Sarah – supervisor)***

### Balancing the risk of “missing something” against the risks of iatrogenic harm

Participants described patients who were frustrated by the lack of physical diagnosis for their distressing symptoms. They felt these patients had difficulty understanding and accepting the uncertainty of medically unexplained symptoms. However, they also described the harm that can come from remaining focused on a physical cause for ongoing symptoms.

*“She saw a neurologist who, much to his credit, had just said, "Look, I don’t think there’s very much organic going on here,” … But after that it was just a sort of a spiral, it was like that cascade effect, where someone sees a specialist, and because the thing is not then within the specialty for which they are trained, they don’t feel able to exclude organic pathology, and will therefore make a referral …we had two neurologists, an ophthalmologist, a neurosurgeon, a psychologist, vascular surgeon, endocrinologist, rheumatologist and cardiologist! I felt guilty about what was happening, but it was also to some extent, I felt kind of out of my control…And there was this lack of ability to say, “Look, we need to stop now.” And then the final straw was when she got admitted to hospital by one of the local surgeons for a leg ulcer and was in there for just months. Really, she should have come home. The whole medicalisation of her internal distress was really strongly embedded as a result.”****(Warren – supervisor)***

On the other hand, participants stressed the importance of taking physical symptoms seriously to establish trust.

*“I would always do a full examination… Kind of get him on side. ..once he’d been taken seriously, I wasn’t judging him or laughing at him or anything, he could see I was worried about him. He became quite open about psychological care.”****(Beth – registrar)***

The anxiety experienced by the patient was often paralleled by the anxiety felt by the doctor, particularly in the case of the registrars. Warren describes this worry about “missing something” as a “niggling biomedical doubt”.

*“you probably haven’t got the luxury of shifting into something chronic, because there’s always the possibility that one of these vague undefined symptoms might turn into cancer”****(Ellen – registrar)***

*“I thought, seventy-five percent plus, it would be anxiety related; maybe about twenty-five percent there was a potential chance it could be something else. I didn’t want to miss something else.”****(Daniel – registrar)***

### Searching for illness explanatory frameworks

#### Having a name for the illness

Several participants talked about the importance of having a name for an illness. The name not only represents the social value attached to a “legitimate” illness, it also means patients have a way of accessing services. For Jonathan, the lack of a name meant his patient was abandoned by tertiary services and was left without support, or a framework to make sense of her suffering.

*“It came to a point where the family were getting more and more desperate, and the father said, “I used to respect doctors, before this whole process, and now I couldn’t think of a profession I respect less. The arrogance and isolation that we have felt from this whole process is devastating.”****(Jonathan – supervisor)***

Participants talked about the process of exclusion: where patients were told what diseases they didn’t have, but had no framework for the illness they were experiencing.

*“I think a lot of the time the complaints she was presenting with would be taken seriously, but it would be that just single complaint and so she’d come to hospital and have chest pain and so they do an ECG and she’d have troponins done and they were negative and then she’d have a stress test and that would be negative and so, well, “No, your heart’s fine.” But she still hadn’t been given a diagnosis as to why she’d had the chest pain. It was just, “Your heart’s fine.” And they just hadn’t gone on to the next step.”****(Victoria – supervisor)***

Participants in the study were reluctant to use diagnostic terms that had acquired stigma, so few of the GPs in the study used the psychiatric classifications for medically unexplained symptoms.

*“I think historically that you know, there’s hysteria and conversion disorder and all that kind of, the old, all the old names sort of just, leave a bad taste.”****(Leon – supervisor)***

*“Whether you voice it to them or not, it’s not something you want to think of on their behalf easily…. it does give me some negative emotions about them, about the relationship that we’ve had….and about my effectiveness and ability in that consult….its almost like I’ve consigned them to something, you know, I’ve consigned them to the scrap.”****(Xavier S)***

Instead, most of the experienced GPs used informal terms or stories to describe and explain symptoms. Most of the GPs had a way of linking emotional issues with physical symptoms, usually using metaphors or models to describe the connection in a way that was careful to acknowledge that symptoms were “real” and not “all in your head”.

*“the explanation that physical symptoms can be produced as a result of mental problems is often very comforting to patients: it can be a comforting wrap around things for both of you”****(Yvonne – supervisor)***

### Managing the chaotic consultation

#### Managing patient expectations when the consultation does not provide a diagnosis and a remedy

*“there’s chaos in the consult… because there’s chaos in the patient”****(Ellen – registrar)***

The consultation around medically unexplained symptoms follows an unfamiliar path for patients. Many participants commented that patients expected a diagnosis and remedy, and without it, the consultation felt chaotic. The supervisors managed this difficulty by making the process of the consultation overt: establishing a framework for patients.

*“I think disorientation is something that is anxiety producing, plus, plus, plus. The unknown is scary. And so, if people know the subtext of what’s going on: the reason why he’s talking about this now is because he’s going to get to that other point that I want to talk about later, it helps”****(Jonathan – supervisor)***

Jonathan described this framework as “getting all the baggage on the table” before negotiating what items of the patient’s and the GP’s agenda could be covered in the consultation.

*“So at the beginning of the consultation, someone comes in with baggage and they go, “I’ve got this and I’ve got that” So that’s what they come in with, their baggage. And you’ve got to let that be put on the table. And defining what is on the table is a critical moment in the consultation. And I talk a lot about this with registrars. To get to the “Is there anything else?” question as soon as possible. And get to a “No,” to that question as soon as possible. “Have you got any other problems? Anything, any other issues?” (laughing) “Fred? No more? Right? Done? Lock it in, Freddy! Right!” And then I go on to my agenda.”****(Jonathan- Supervisor)***

Despite these strategies, it was clear that many experienced supervisors encountered consultations that were chaotic.

*“So it was like this kind of cloud of things you had to wade through to even move forward at all. Consultations were always long and they always went nowhere. Despite being a relatively experienced GP who I thought usually could sort of cut through the chaff pretty quickly, but, um, she beat me! It’s almost like a black hole, isn’t it?”****(Warren – supervisor)***

Several participants commented that they needed to help patients “shift gear”: transitioning from a focus on cure, to assisting the patient to cope. This shift was often uncomfortable for doctors and patients.

*“that moment of shifting gear, when you go from chasing down that elusive physical diagnosis to shifting gear and realising that this is a chronic complex ongoing thing that involves an awful lot of support…it’s like your role shifts into a different space.”****(Daniel-registrar)***

### Teaching and learning

#### Learning professional values

Supervisors discussed the importance of teaching registrars to value patients, and accept their ethical responsibility to care for them. Many of the supervisors were quite forthright in describing the role of a GP.

*“This is a person, and even though they’re a bit shabby, sweaty, whatever, doesn’t matter whether you want them living next door to you or not, they’re a person, so you’ve got to work out their context and how to help them negotiate the system. Otherwise, you’re in the wrong job. Get a job as a pathologist.”****(Oscar – supervisor)***

However, some of the registrars felt uncomfortable providing support and validation: they were concerned they were encouraging an unhealthy dependence. Ellen, one of the registrars, described a relationship in which she had needed to become

*“less open and warm… because I think he really enjoys it too much and it’s not helpful for him… And I think I’m not the best person to be giving him that kind of supportive therapy anymore.”****(Ellen - registrar)***

Registrars found it quite difficult to describe what it meant to experience a relationship as ‘unhelpful’, far less to predict when this might occur. They expressed the need to learn more about this aspect of clinical practice from their supervisors:

*“… how they separate from their patients, and how much they take on, and where they draw the line and where they put their boundaries, and why they will see this patient at 6 o’clock on a Friday, but they won’t see that patient at 6 o’clock on a Friday.”****(Anna- registrar)***

Some supervisors felt this discomfort arose because GP registrars were expected to make their own independent ethical choices around patient care, and this was confronting.

***“****this is probably the first time they’re making their own ethical choices about what they’ll do with patients. Because in hospitals, you know, there’s protocols and there’s teams and everyone works together to decide how much social goods a patient will have, how long they’ll stay in hospital, what services we’ll offer. In general practice, you’re making that choice on your own, and often for registrars, this is the first time where the buck really stops with them. And it can be quite challenging.”****(Quentin – supervisor)***

Some of the registrars explicitly mentioned the way patients with medically unexplained symptoms were not valued in the hospital context. Anna, for instance, commented that it was culturally accepted for medical staff to focus their energies on other patients.

*“The ‘heart sink’ was never there because if you didn’t like the next person on the list then you didn’t see them (laughs) and you got to know the ones that you didn’t want to see.”****(Anna, Registrar)***

The supervisors did not describe specific models of care to assist registrars manage patients with medically unexplained symptoms. Instead, they focussed on building insight and establishing professional expectations; being explicit that the care of these patients was a legitimate and important part of GP “work”. Part of that discussion included validating the registrar: explaining that these patients can be challenging to manage.

*“I think just talking to them about the fact that, “Oh, yes, look, did you look through the notes? There’s ten years’ worth of this in here!” That’s often associated with a big sense of relief on the registrar’s part that it’s not just them, that you know, this is what’s happening with the patient.”****(Warren- Supervisor)***

### Learning about legitimate “work”

The participants in the study felt that patients needed to be valued as legitimate patients with legitimate illnesses and the supervisors expressed the importance of teaching this value to their registrars. There was a parallel experience: where the doctors felt their work in caring for patients with medically unexplained symptoms was not valued. Supervisors commented that registrars had to leave behind the value that “good” medicine always involved a clear diagnosis and a cure.

*“A diagnosis provides a conclusion to the process and a justification that the doctor has done a good job. And a non-diagnosis potentially makes the registrar feel that the reason they’ve got a non-diagnosis is because they are fundamentally incompetent, rather than the fact that there isn’t a diagnosis at all”****(Quentin – supervisor)***

Registrars struggled to define “good doctoring”; particularly with these patients. Ellen, a remote area doctor, described a complex case; a homeless man with multiple serious physical illnesses, who had a vulnerable personality and a series of complex social needs. She describes the transition in her understanding of “good doctoring” over the course of her first general practice term.

*“well I think initially, I thought the important thing to be a Good Doctor for that patient was to offer the continuity, and to be comprehensive. To ensure that there were no loose ends, and everything was followed up, and investigated and managed. But now I reckon for him, a Good Doctor would be someone who knew when to say no, and stop investigating, and just try and think more creatively about strategies to help him deal with where his life is at now.”****(Ellen – registrar)***

Supervisors shared their own diagnostic and management decisions. However, there was a balance: supervisors felt registrars needed to establish their own professional practice and boundaries. They acknowledged it may be difficult for some registrars to develop the skills and capacity to undertake this sort of medical work. Several supervisors commented that registrars needed to learn how to tolerate uncertainty and manage “unfixable” suffering, and that these skills took time and maturity to develop.

*“I think one of the traps we fall into is the same trap the registrar falls into. We want to change the registrar into something. And I actually think it’s something that can’t be taught. It has to be a growing awareness. It has to bubble up…the question that’s fascinating to me, is ‘What are they like in the presence of unfixable suffering?’ “****(Robert – supervisor)***

Robert’s comment was echoed by several supervisors; the sense that these skills are acquired through reflective experience, rather than through the acquisition of particular models or methods. They did stress the importance of sharing care of these patients, including observing consultations.

*“just offering to call me in when the patient’s in next; that’s quite powerful. The patient doesn’t necessarily like that but it is a way of kind of bringing things back to earth again, it’s sort of regrounding the whole scenario. Okay, these are the ground rules. This is what we do. This is how we move forward.”****(Warren- Supervisor)***

### Supporting the registrar’s role in providing care

Registrars struggled with understanding what they should legitimately provide for patients. They expressed strong personal values around caring for patients, but worried that their inexperience interfered with their ability to provide good care. Finding the balance between good quality, meticulous care and an obsessive and unhelpful need for certainty was a difficult skill for many registrars to acquire.

*“I’ve had registrars in the past that have been overly vigilant with patients and sort of encouraged or enabled dependence by their hypervigilance.”****(Leon – supervisor)***

Warren described the limits of the registrar-patient relationship. Given registrars have a temporary placement, he feels that their roles should be more circumscribed and different to the roles played by the senior doctors.

*“the registrar involvement is actually seen as an adjunct or as a supplement or a complement, where the relational anchor is with the principal.”****(Warren – supervisor)***

While medically unexplained symptoms can present in one consultation, in this study, the cases discussed were patients with chronic and complex needs, and so the importance of ongoing care and support was emphasised by the participants. Supervisors discussed the importance of shared consultations, to ensure continuity of care. They also discussed the importance of careful handover between registrars to balance the patient’s need for continuity, and the registrars’ need to learn the care of patients with chronic illness.

### Learning explanatory frameworks

The registrars struggled to explain the link between emotional and physical symptoms.

*they’re worried about actually saying to somebody, I don’t think you’ve got an organic or physical problem. Or they don’t know when to say it or how to bring it up.”****(Leon – supervisor)***

There was a sense for some of the registrars that they lacked models and metaphors to make sense of medically unexplained symptoms.

***“****How would you explain that? I’d probably have to think very fast … you know how, over time you learn the lines? I don’t have that line yet.”****(Beth – registrar)***

Supervisors also discussed the importance of an open mind: “listening to the illness, but keeping an eye out for the disease”. They felt this was a skill that was refined over time with experience, so although they suggested strategies to manage the uncertainty (shared consultation, consultation review) they recognised that this was a difficult skill to teach.

*“You’ve always got to keep an open mind about the physical side of things. I’ve been treating someone psychologically for years and I’d end up missing physical things because we’d put down their symptoms, their physical symptoms down to psychological causes. Trying to get that balance between under-investigating and over-investigating; I don’t think there’s an easy rule-book for that, it’s one of those things that registrars need to get a feel of, over time.”****(Sara- Supervisor)***

### Learning to manage the chaotic consultation

#### Finding a consultation structure when there is no obvious diagnosis or treatment

Some supervisors observed that registrars often needed to offer more direction in their consultations. They described “shifting from content to process”: helping registrars to understand and manage the consultation process as a core clinical task.

*“Generally they don’t realise the degree of control that they’re exerting over the consultation. So the first thing is creating awareness that what they are actually doing is creating the direction the consultation is going to go. Once they recognise that they have a choice, I think that is the first step in them deciding how far down each line they go.”****(Michael – supervisor)***

The supervisors had a range of consultation structures available to them, and felt more able to alter their approach according to the needs of their patients. They talked about “signposting”: giving the registrars a sense of the stages of a consultation. One supervisor commented that is like sharing the “masterslide” in a presentation- providing an overview and a structure that registrars are able to use to structure the consultation effectively. Several mentioned explicitly attending to a personal sense of frustration as a marker that the consultation may be becoming unhelpful or disordered. Supervisors and registrars spoke a lot about boundaries, although they were often not explicit in describing why and when a boundary needed to be in place. They did talk about scheduling consultations in a regular, predictable and time limited way to reduce the chance of patients presenting in crisis.

### A theoretical model for the consultation

In this study, it emerged that the consultation process around medically unexplained symptoms is prone to being dis-ordered. The literature suggests that effective consultations involve co-constructed meaning, with patients drawing on illness schema and doctors drawing on disease schema [22]. These schemas evolve into co-constructed frameworks for the presenting illness. Frameworks then become part of the discourse of the respective social and professional communities and continue to shape schemas around illness and disease.

**Figure 1:**
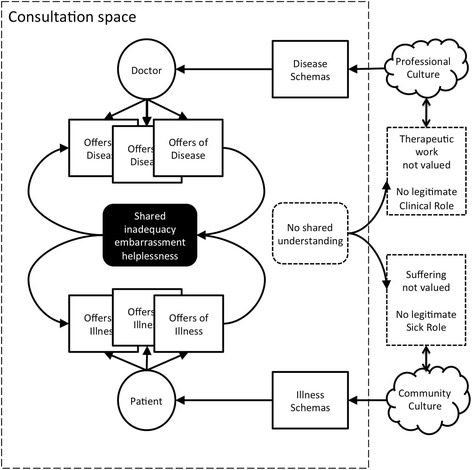
**The consultation around medically unexplained symptoms.**

Figure 1 models the process that the doctors described when they were unable to develop a shared framework. Repeated presentations can cause escalating frustration for doctor and patient. Without a name for the illness, the patient is unable to feel they have a legitimate illness and the doctor may feel that the consultation is not a legitimate use of their time. Understandably, patients then continue to seek an “answer” for their suffering through repeated presentations and progressively more challenging exchanges: the “duet of escalating antagonism” described by Kleinman [20]. Opinions shared in the consultation shape illness and disease schemas in the respective personal and professional communities, reinforcing the cultural barriers around medically unexplained symptoms.

**Figure 2:**
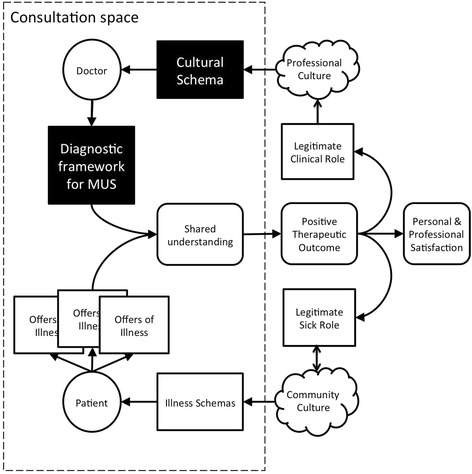
**A model consultation using helpful schemas for medically unexplained symptoms.**

The experienced GPs and some of the registrars in this study managed these consultations differently. They had their own professional culture which accepts medically unexplained symptoms as real and important experiences. There are a number of disease schemas around these symptoms, including formal categorical diagnoses, such as somatisation, but there are also storied frameworks. GPs talked about symptom presentations overlying stories of patients with complex backgrounds incorporating childhood trauma and multiple psychosocial stressors. They described patients who lacked the resources to make sense of their illnesses, or manage their distress. Figure 2 models how they bought these schemas into the consultation to reach a shared understanding of the problem without necessitating a physical diagnosis. This approach breaks the looping effects represented in Figure 1.

## Discussion

Supervisors and registrars in this study expressed a sincere desire to care for patients with medically unexplained symptoms, even though they acknowledged that the therapeutic relationship could be difficult. This is consistent with previous work around the care of patients with medically unexplained symptoms [30]. Supervisors also expressed a strong commitment to establishing professional expectations: teaching registrars that the management of medically unexplained symptoms was legitimate general practice “work”. Part of this work involved understanding that diagnosis is not always possible: it is a tool rather than *“a justification that the doctor has done a good job.”*

There was a strong sense in this study that registrars were undergoing a cultural shift, as well learning new skills. The registrars commented that these patients were not valued in the hospital setting, and so they needed to reorient their thinking to adopt a new cultural role as primary health care providers. While it was not explicitly addressed in this study, several supervisors commented on registrars who struggled to make this cultural transition. If supervisors are enabling this cultural shift, then it is important to explicitly address the importance of role modelling, and training in situated professional behaviours during supervisor professional development. These patients, with their medical, psychological, organisational and interactional complexities, offer an ideal opportunity for supervisors and registrars to explicitly discuss the important professional transition to primary care.

These consultations can be overwhelming and, consistent with previous work in the area, this study demonstrates how GPs struggle to avoid distancing themselves to manage their own difficult feelings [37,38]. Supervisors stressed the need to allow these skills to “bubble up”: they felt that observation and experience were necessary to enable the acquisition of appropriate strategies and techniques. While there are many programs in place to support the management of difficult interpersonal interactions (eg Balint groups [64–67]), this study supports the need for one on one supervision and mentorship. These skills are difficult, contextualised and personal, and supervisors and registrars recognised that they need to be acquired through reflective professional practice.

Another difficult skill involves risk management. Registrars need to learn to tolerate uncertainty and to manage the risk of iatrogenic harm. This includes reducing the risk of entrenching an unhelpful focus on physical symptoms, the iatrogenic potential of the consultation itself [68]. Supervisors discussed the importance of attending to both physical and psychological symptoms: “listening to the illness but keeping an eye out for the disease”. GPs are in a unique position to manage holistic care, but registrars may benefit from formally attending to and discussing physical, psychiatric and psychosocial formulations of illness separately in these cases, to minimise the risk that significant illness is overlooked.

The supervisors described a few educational strategies for assisting registrars in their “good doctoring”. Like the patients they described, they recognised that registrars needed validation and individualized support. They described strategies to help learners uncover their own difficulties and identify their own solutions. During supervision, they shared consultations and observed interactions, encouraging reflection and providing support. In particular, they encouraged a “shift from content to process”: encouraging registrars to see the process of the consultation as an important and “learnable” skill. Interestingly, no supervisors mentioned existing models of care for patients with medically unexplained symptoms, such as reattribution. Given the deep experience of the supervisor cohort, this may reflect an opportunity for supervisor education and professional development.

Supervisors shared a bank of metaphors, heuristics and models. Most of these models were idiosyncratic, with none of the participants mentioning diagnostic classifications of medically unexplained symptoms unless specifically asked. The reluctance to use “labels”, which were perceived to be stigmatising and unhelpful, restricts the ability of clinicians to think about medically unexplained symptoms in a structured way. Again, there is opportunity for specific supervisor educational development in this area, considering the different roles of diagnosis as a way of understanding illness for the clinician, sharing understandings between clinicians and using diagnosis as a “label” for the patient.It may be helpful to consider how diagnosis can be reframed to meet each requirement.

### Parallels between the patient and registrar experience

When patients present with medically unexplained symptoms, there are parallels between the patient and the doctor experience. Patients with illness, but no obvious disease challenge personal and professional values: medically unexplained symptoms are not valued highly in either community or medical culture. Just as there are questions for the patient around what is considered legitimate illness, there are questions for the doctor around what is considered legitimate medical “work”. Both patients and doctors can struggle to manage the uncertainty inherent in these illnesses, and can become committed to “chasing down” an elusive diagnosis, often with iatrogenic consequences. Doctors and patients can become frustrated with the chaotic consultation structure, and the challenging interpersonal interactions that often characterise these illnesses.

Table 2 shows some of these parallels, and the potential responses that GPs can make with patients, and supervisors can make with registrars. Without addressing these parallels, there is the potential for registrar and patient concerns to feed off each other, creating an unhelpful “vicious cycle”: Kirmayer’s looping effects [39]. A classic example of this dynamic is when a registrar’s anxiety about “missing something serious” can exacerbate a patient’s anxiety that they have a serious physical illness no-one has managed to detect.

**Table 2:** **Parallels between the patient and registrar experience**

	**Patient experience (based on the existing literature)** **[** 11,12,20,37,39,64,68–95 **]**	**Registrar experience**	**Potential response to the patient**	**Potential response to the registrar (by the supervisor)**
**The consultation process**	Feels chaotic because the doctor cannot offer me an organic cause for symptoms	Feels chaotic because I cannot identify a diagnosis and evidence-based guideline	Open communication and explanation about the process	Discussion around models of the consultation process
**Patterns of attention and avoidance**	Emphasis on physical symptoms allows me to be “taken seriously”.	Missing an organic diagnosis would be a serious error: I must attend carefully to physical cues to avoid this risk	Accepting and attending to psychosocial issues	Encouraging empathic connection regardless of symptoms
**Illness explanatory frameworks**	Cannot find an illness explanatory framework or explanatory frameworks are complex, chaotic or contradictory	Cannot find a disease explanatory framework or explanatory frameworks are complex, chaotic or contradictory	Sharing explanations beyond a disease model. May involve narratives and metaphors.	Sharing understanding through explicit and/or implicit models. May involve case studies and stories
**The battle for legitimacy**	Perception that doctors become frustrated because I am not “getting better”	Uncertainty as to whether this is a good use of my time: am I just creating dependence?	Recognising and respecting the patient’s suffering and their right to care	Helping registrar to manage suffering in the absence of disease
**Interpersonal relationships**	My suffering is not recognised by others	My efforts to help are not valued by others	Recognition and reassurance	Recognition and reassurance

This study demonstrates how supervisors draw on their experience of patient illness and their resultant metaphors, models and frameworks to “make sense” of complex and distressing symptoms. To learn these skills, registrars need supervisors to articulate their reasoning, and be open about the interpersonal challenge of managing difficult therapeutic interactions. These skills are not easily learned, or taught.

### Strengths and limitations of the study

This study focussed on the GPs of patients with medically unexplained symptoms. Interviews explored a diverse group of patients that reached beyond categorical psychiatric disorders. The study also engaged a highly diverse group of GPs across Australia, who provided rich data. The constructivist grounded theory methodology of this study facilitated the development of concepts and frameworks iteratively which enriched both the data and the analysis.

Further study in this area could involve interviewing doctors and their patients over time. This could explore differences in understanding and experience between doctors and their patients, and highlight how diagnostic thinking develops. Observation of medical behaviour, rather than just reflection on diagnostic thinking would also enrich our understanding of this complex area.

The interviews revealed strong feelings in the GPs, and although this study focussed on the way they made sense of the patient’s presentation, further work could explore the discomfort experienced by GPs when managing situations of high uncertainty. This was particularly interesting in the GP registrars who were navigating the transition between tertiary and primary care.

A broader sampling frame may also enrich understanding. In this study, participants were drawn from an expert sample who were experienced in reflecting and communicating their clinical thinking and behaviour. It may be helpful to explore the attitudes of a broader range of GPs, particularly those who do not identify an interest in mental health. Conversely, there may be benefit exploring the thinking of a range of GPs who identify interest and competence in psychotherapy. Although grounded theory generated a rich methodological framework for this study, it would also be interesting to explore the experience of doctors and patients in this area using phenomenological or narrative perspectives.

This study focussed on the reasoning of doctors. There have been many studies around the lived experience of chronic illness, but few focussing on the way “unexplained” illness changes this experience. With the ready availability of patient education materials and public forums online, this experience increasingly involves patients constructing their own explanations external to the medical world. There is a role for qualitative research around shifting illness meanings, and the way they affect patients within their own social worlds. Such research could inform better holistic, patient-centred care.

The model of the consultation proposed in this study needs to be validated with observational data from actual consultations. Registrars already record consultations for later discussion with their supervisors. It would be interesting to examine consultations with patients with medically unexplained symptoms and refine the proposed model. It would also be interesting to take several of these recordings and ask supervisors how they would debrief a registrar, to explore teaching strategies utilised in this difficult area of practice.

## Conclusion

Patients with medically unexplained symptoms commonly present in general practice and many experience profound suffering. This study supports the existing literature, suggesting that GPs face significant challenges managing patient care. However, this study also suggests that the experience of patients and the experience of their doctors have interesting and challenging parallels. A key shared experience is the desire for a concrete diagnosis to make sense of suffering and the discomfort that comes from living with uncertainty.

These patients confront GPs with uncomfortable feelings. They challenge them to think about what it means to be a doctor and what it means to be ill. Registrars often manage these patients in a conceptual void, without words to make sense of their distress and without tools to mitigate their suffering.

For registrars, these patients represent an opportunity for learning that is broad and multifaceted. However, if learning is to occur, registrars need to feel safe to discuss issues that can be personally and professionally confronting. This study suggests that supervisors have rudimentary frameworks which are idiosyncratic. They rely on coping strategies based on personal experience, and teach registrars through experiential strategies. Further emphasis on cognitive and emotional reflection may help registrars learn to manage these patients more effectively.

Like an old-fashioned embroidery sampler, patients with medically unexplained symptoms require a series of new “stitches” that the registrar needs to master. These include difficult consultation dynamics, confronting personal feelings, extensive care coordination and advocacy and questions around personal and professional values. Because of this, patients with medically unexplained symptoms can present an unparalleled opportunity for learning. Supervisors can facilitate this process by opening these discussions and making their own diagnostic and management strategies overt. By discussing the difficult feelings these patients engender, they can also open up a discussion of values and professional roles in this challenging area of practice.
